# Comprehensive evaluation of treatment and outcomes of low-grade diffuse gliomas

**DOI:** 10.1371/journal.pone.0203639

**Published:** 2018-09-20

**Authors:** Catherine R. Garcia, Stacey A. Slone, Thomas Pittman, William H. St. Clair, Donita D. Lightner, John L. Villano

**Affiliations:** 1 Markey Cancer Center, University of Kentucky, Lexington, Kentucky, United States of America; 2 Division on Cancer Biostatistics, University of Kentucky, Lexington, Kentucky, United States of America; 3 Department of Neurosurgery, University of Kentucky, Lexington, Kentucky, United States of America; 4 Department of Radiation Oncology, University of Kentucky, Lexington, Kentucky, United States of America; 5 Department of Neurology, University of Kentucky, Lexington, Kentucky, United States of America; 6 Department of Medicine, University of Kentucky, Lexington, Kentucky, United States of America; Centre Hospitalier Sainte Anne, FRANCE

## Abstract

**Background:**

Low-grade gliomas affect younger adults and carry a favorable prognosis. They include a variety of biological features affecting clinical behavior and treatment. Having no guidelines on treatment established, we aim to describe clinical and treatment patterns of low-grade gliomas across the largest cancer database in the United States.

**Methods:**

We analyzed the National Cancer Database from 2004 to 2015, for adult patients with a diagnosis of World Health Organization grade II diffuse glioma.

**Results:**

We analyzed 13,621 cases with median age of 41 years. Over 56% were male, 88.4% were white, 6.1% were black, and 7.6% Hispanic. The most common primary site location was the cerebrum (79.9%). Overall, 72.2% received surgery, 36.0% radiation, and 27.3% chemotherapy. Treatment combinations included surgery only (41.5%), chemotherapy + surgery (6.6%), chemotherapy only (3.1%), radiation + chemotherapy + surgery (10.7%), radiation + surgery (11.5%), radiation only (6.1%), and radiotherapy + chemotherapy (6.7%). Radiation was more common in treatment of elderly patients, 1p/19q co-deletion (37.3% versus 24.3%, p<0.01), and tumors with midline location. Median survival was 11 years with younger age, 1p/19q co-deletion, and cerebrum location offered survival advantage.

**Conclusions:**

Tumor location, 1p/19q co-deletion, and age were the main determinants of treatment received and survival, likely reflecting tumor biology differences. Any form of treatment was preferred over watchful waiting in the majority of the patients (86.1% versus 8.1%). Survival of low-grade gliomas is higher than previously reported in the majority of clinical trials and population-based analyses. Our analysis provides a real world estimation of treatment decisions, use of molecular data, and outcomes.

## Introduction

Low-grade gliomas have a wide variety of histologic and molecular features corresponding to a grade II in the World Health Organization (WHO) Classification of Central Nervous System Tumors[1]. This group includes astrocytomas, oligodendrogliomas, and oligoastrocytomas. However, evidence of molecular genetic analyses demonstrates that the vast majority of tumors previously classified as oligoastrocytomas have a genetic profile typical of either diffuse astrocytoma or oligodendroglioma, with few true cases of oligoastroctyomas[1]. Therefore this category is now designed as NOS in the 2016 classification [2], and its use is expected to decrease.

Low-grade gliomas are most common in young adults between 35 and 44 years of age[3]. They are a slower growing group of tumors, but a subgroup can be fast growing; still their prognosis is favorable compared to high-grade gliomas. However, most low-grade gliomas eventually transform into high-grade gliomas, resulting in debate in determining the first course of treatment, the time and aggressiveness of surgery, and the role of adjuvant treatment. Watchful waiting until progression may be an acceptable option in selected patients, however surgical resection often results in improved outcomes and symptom control, particularly tumor-related epilepsy[4–6], which is of especial interest due to the higher rate of seizures in isocitrate dehydrogenase (IDH) mutant tumors[7].

Since 2016 molecular markers have been included in the classification[1]. This has translated into treatment decisions; presence of IDH mutation, 1p/19q co-deletion, ATRX expression, and TERT promoter mutation are used in diagnosis and provide prognosis estimates[8, 9]. Other factors such as older age (>40 years), incomplete bulk tumor resection, or having an unfavorable molecular profile, such as IDH wild type, are considered in treatment decisions for possible radiation and chemotherapy[10–13]. An established standard of care has not been defined, and treatment strategies often differ among physicians. Our analysis describes the prognosis and therapeutic patterns of care for diffuse gliomas in American College of Surgeons Commission on Cancer (CoC)-accredited hospitals in the United States using the National Cancer Database (NCDB).

## Methods

NCDB is a joint program of the American College of Surgeons Commission on Cancer (CoC) and the American Cancer Society that collects cases from over 1,500 CoC-accredited hospitals. With over 34 million cases, it is the largest cancer database in the United States. Data collection methods, characteristics of participating hospitals and patients, and assessment of data quality have been described elsewhere, and has been used extensively in treatment description of primary brain tumors[14, 15].

We used the 2015 NCDB brain/central nervous system participant user file, which includes cases from 2004 to 2015. Institutional Review Board approval was obtained as exception as determined by the University of Kentucky.

Cases of diffuse glioma were identified using the International Classification of Disease for Oncology (ICD-03) histology codes 9380–9382, 9400, 9401, 9410, 9411, 9420, 9421, 9424, 9425, 9431, 9450, 9451, 9460. Patients with diagnosis of WHO grade I, III, IV, or not reported, and patients younger than 18 years were excluded from the analysis.

Demographic variables that were analyzed include age at diagnosis, sex, race, Hispanic origin, primary payer status, median income, education status, and urban/rural residence. Living area, as determined by the zip code of the patient recorded at the time of diagnosis was used to classify patients as urban or rural, as defined by the United States Department of Agriculture Economic Research Service[16]. Charlson-Deyo score[17] was used to assess patient comorbidities and was categorized as 0, 1, 2, or ≥ 3, as available in NCDB. Tumor location was defined as midline (brainstem, spine, and ventricles not otherwise specified), cerebrum (cerebrum and lobes), or other (including meninges, brain, and nervous system not otherwise specified). Education was assessed by the percentage of non-high school graduates in the patient zip code at the time of diagnosis (≤ 7%, 7–12.9%, 13–20.9%, ≥ 21%).

Disease characteristics included in our analyses were primary site location, histology group, chromosome 1p, and chromosome 19q loss of heterozygosity. Histology groups were divided into astrocytic (9400, 9401, 9410, 9411, 9420, 9421, 9424, 9425), oligodendroglial (9450, 9451, 9460), and mixed (9380, 9381, 9382) as NCDB uses the WHO 2007 classification. 1p/19q co-deletion status was available for cases diagnosed after 2010. Co-deletion was defined as having both chromosomal arm deletions as reported in the Collaborative Stage Site-Specific Factors by NCDB, cases negative for both chromosome deletions were defined as negative, and patients with only one deletion reported were considered incomplete. Treatment received was assessed as first course of treatment at any CoC facility, as previously described by our group[18].

### Statistical analysis

Chi square was performed for categorical variables. Age groups cutoff were determined based on historical reports[10, 11] and increased mortality cutoffs using area under the curve. Survival and risk of mortality was assessed as previously described by our group using Kaplan Meier and Cox proportional hazards models[18]. The level of statistical significance was set at 0.05 for all tests conducted, and all analyses were performed with SAS software version 9.4 (SAS Statistical Institute, Cary, North Carolina).

## Results

A total of 13,621 cases were identified. The median age at diagnosis was 41 years (range: 18–90) and 56% were male. Race was collected for 98.5% of the patients and was grouped in white, black, and others. The most common race was white (88.5%), followed by black (6.1%), and other races (3.9%). Hispanic origin was available for 93.6% of the patients, with 7.5% identified as Hispanic. Hispanic origin was more common in younger age groups. Younger patients had a higher proportion of blacks and other races (Table 1), that may represent demographic trends in the United States, independent of tumor biology. Eighty-six percent had a Charlson-Deyo score (measurement of co-morbidities) of zero, while 1% had a score ≥3. Charlson-Deyo score increased with increasing age but did not vary by histological groups or molecular determinants (Table 2 and Table 3).

**Table 1:** Characteristics of the study population, by age group.

		18–40 N = 6656	41–60 N = 5113	≥60 N = 1852	All N = 13621	p-value
**Gender**	Male	3767 (56.6%)	2890 (56.5%)	973 (52.5%)	7630 (56.0%)	< .01
	Female	2889 (43.4%)	2223 (43.5%)	879 (47.5%)	5991 (44.0%)	
**Race**	White	5798 (87.1%)	4568 (89.3%)	1685 (91.0%)	12051 (88.5%)	< .01
	Black	438 (6.6%)	302 (5.9%)	97 (5.2%)	837 (6.1%)	
	Other	315 (4.7%)	166 (3.2%)	52 (2.8%)	533 (3.9%)	
	Unknown	105 (1.6%)	77 (1.5%)	18 (1.0%)	200 (1.5%)	
**Hispanic**	Unknown	407 (6.1%)	335 (6.6%)	126 (6.8%)	868 (6.4%)	< .01
	No	5644 (84.8%)	4447 (87.0%)	1640 (88.6%)	11731 (86.1%)	
	Yes	605 (9.1%)	331 (6.5%)	86 (4.6%)	1022 (7.5%)	
**Tumor location**	NOS	997 (15.0%)	885 (17.3%)	376 (20.3%)	2258 (16.6%)	< .01
	Cerebrum	5430 (81.6%)	4071 (79.6%)	1385 (74.8%)	10886 (79.9%)	
	Midline	229 (3.4%)	157 (3.1%)	91 (4.9%)	477 (3.5%)	
**Charlson-**	0	6040 (90.7%)	4311 (84.3%)	1308 (70.6%)	11659 (85.6%)	< .01
**Deyo**	1	456 (6.9%)	573 (11.2%)	375 (20.2%)	1404 (10.3%)	
**Score**	2	130 (2.0%)	175 (3.4%)	121 (6.5%)	426 (3.1%)	
	≥3	30 (0.5%)	54 (1.1%)	48 (2.6%)	132 (1.0%)	
**Facility**	Unknown	6312 (94.8%)	0 (0.0%)	0 (0.0%)	6312 (46.3%)	< .01
**Type**	Community	143 (2.1%)	2252 (44.0%)	963 (52.0%)	3358 (24.7%)	
	Academic	201 (3.0%)	2861 (56.0%)	889 (48.0%)	3951 (29.0%)	
**Living area**	Unknown	215 (3.2%)	205 (4.0%)	83 (4.5%)	503 (3.7%)	< .01
	Metro	5441 (81.7%)	4075 (79.7%)	1425 (76.9%)	10941 (80.3%)	
	Rural	1000 (15.0%)	833 (16.3%)	344 (18.6%)	2177 (16.0%)	
**Treatment**	Unknown	413 (6.2%)	265 (5.2%)	79 (4.3%)	757 (5.6%)	< .01
	None	432 (6.5%)	416 (8.1%)	261 (14.1%)	1109 (8.1%)	
	RT+CT+S	604 (9.1%)	658 (12.9%)	193 (10.4%)	1455 (10.7%)	
	RT+CT	280 (4.2%)	402 (7.9%)	232 (12.5%)	914 (6.7%)	
	RT+S	680 (10.2%)	654 (12.8%)	235 (12.7%)	1569 (11.5%)	
	CT+S	426 (6.4%)	393 (7.7%)	77 (4.2%)	896 (6.6%)	
	S only	3397 (51.0%)	1797 (35.1%)	464 (25.1%)	5658 (41.5%)	
	RT only	243 (3.7%)	343 (6.7%)	248 (13.4%)	834 (6.1%)	
	CT only	181 (2.7%)	185 (3.6%)	63 (3.4%)	429 (3.1%)	
**Insurance status**	Not Insured	585 (8.8%)	322 (6.3%)	40 (2.2%)	947 (7.0%)	< .01
	Private	4636 (69.7%)	3939 (77.0%)	570 (30.8%)	9145 (67.1%)	
	Medicaid	998 (15.0%)	420 (8.2%)	51 (2.8%)	1469 (10.8%)	
	Medicare	162 (2.4%)	227 (4.4%)	1144 (61.8%)	1533 (11.3%)	
	Other Gov’t	140 (2.1%)	95 (1.9%)	19 (1.0%)	254 (1.9%)	
	Unknown	135 (2.0%)	110 (2.2%)	28 (1.5%)	273 (2.0%)	

**NOS:** Not otherwise specified; **RT:** Radiation therapy; **CT:** Chemotherapy; **S:** Surgery

**Table 2:** Characteristics of the study population, by histology group.

		Astrocytic N = 6050	Mixed N = 2795	Oligodendroglial N = 4776	All N = 13621	p-value
**Gender**	Male	3381 (55.9%)	1593 (57.0%)	2656 (55.6%)	7630 (56.0%)	0.49
	Female	2669 (44.1%)	1202 (43.0%)	2120 (44.4%)	5991 (44.0%)	
**Race**	White	5307 (87.7%)	2479 (88.7%)	4265 (89.3%)	12051 (88.5%)	< .01
	Black	434 (7.2%)	161 (5.8%)	242 (5.1%)	837 (6.1%)	
	Other	223 (3.7%)	109 (3.9%)	201 (4.2%)	533 (3.9%)	
	Unknown	86 (1.4%)	46 (1.6%)	68 (1.4%)	200 (1.5%)	
**Hispanic**	Unknown	355 (5.9%)	202 (7.2%)	311 (6.5%)	868 (6.4%)	0.14
	No	5234 (86.5%)	2378 (85.1%)	4119 (86.2%)	11731 (86.1%)	
	Yes	461 (7.6%)	215 (7.7%)	346 (7.2%)	1022 (7.5%)	
**Tumor location**	NOS	1185 (19.6%)	460 (16.5%)	613 (12.8%)	2258 (16.6%)	< .01
	Cerebrum	4490 (74.2%)	2270 (81.2%)	4126 (86.4%)	10886 (79.9%)	
	Midline	375 (6.2%)	65 (2.3%)	37 (0.8%)	477 (3.5%)	
**Charlson-**	0	5134 (84.9%)	2441 (87.3%)	4084 (85.5%)	11659 (85.6%)	0.07
**Deyo**	1	653 (10.8%)	251 (9.0%)	500 (10.5%)	1404 (10.3%)	
**Score**	2	204 (3.4%)	73 (2.6%)	149 (3.1%)	426 (3.1%)	
	≥3	59 (1.0%)	30 (1.1%)	43 (0.9%)	132 (1.0%)	
**Facility**	Unknown	2720 (45.0%)	1458 (52.2%)	2134 (44.7%)	6312 (46.3%)	< .01
**Type**	Community	1660 (27.4%)	568 (20.3%)	1130 (23.7%)	3358 (24.7%)	
	Academic	1670 (27.6%)	769 (27.5%)	1512 (31.7%)	3951 (29.0%)	
**Living area**	Unknown	247 (4.1%)	114 (4.1%)	142 (3.0%)	503 (3.7%)	0.96
	Metro	4834 (79.9%)	2237 (80.0%)	3870 (81.0%)	10941 (80.3%)	
	Rural	969 (16.0%)	444 (15.9%)	764 (16.0%)	2177 (16.0%)	
**Treatment**	Unknown	334 (5.5%)	146 (5.2%)	277 (5.8%)	757 (5.6%)	< .01
	None	629 (10.4%)	205 (7.3%)	275 (5.8%)	1109 (8.1%)	
	RT+CT+S	692 (11.4%)	348 (12.5%)	415 (8.7%)	1455 (10.7%)	
	RT+CT	588 (9.7%)	167 (6.0%)	159 (3.3%)	914 (6.7%)	
	RT+S	736 (12.2%)	377 (13.5%)	456 (9.5%)	1569 (11.5%)	
	CT+S	167 (2.8%)	168 (6.0%)	561 (11.7%)	896 (6.6%)	
	S only	2202 (36.4%)	1182 (42.3%)	2274 (47.6%)	5658 (41.5%)	
	RT only	565 (9.3%)	124 (4.4%)	145 (3.0%)	834 (6.1%)	
	CT only	137 (2.3%)	78 (2.8%)	214 (4.5%)	429 (3.1%)	
**Insurance status**	Not Insured	412 (6.8%)	209 (7.5%)	326 (6.8%)	947 (7.0%)	< .01
	Private	3859 (63.8%)	1896 (67.8%)	3390 (71.0%)	9145 (67.1%)	
	Medicaid	661 (10.9%)	329 (11.8%)	479 (10.0%)	1469 (10.8%)	
	Medicare	880 (14.5%)	255 (9.1%)	398 (8.3%)	1533 (11.3%)	
	Other Gov’t	112 (1.9%)	55 (2.0%)	87 (1.8%)	254 (1.9%)	
	Unknown	126 (2.1%)	51 (1.8%)	96 (2.0%)	273 (2.0%)	

**NOS:** Not otherwise specified; **RT:** Radiation therapy; **CT:** Chemotherapy; **S:** Surgery

**Table 3:** Characteristics of the study population, by 1p/19q co-deletion status.

		1p/19q co-deleted N = 901	Non co-deleted N = 1003	Incomplete N = 196	Unknown N = 11521	All N = 13621	p-value
**Age**	Median(years)	37 (18–83)	42 (18–80)	38 (18–79)	41 (18–90)	41 (18–90)	< .01
**Gender**	Male	511 (56.7%)	562 (56.0%)	110 (56.1%)	6447(56.0%)	7630(56.0%)	0.98
	Female	390 (43.3%)	441 (44.0%)	86 (43.9%)	5074(44.0%)	5991(44.0%)	
**Race**	White	798 (88.6%)	902 (89.9%)	172 (87.8%)	10179(88.4%)	12051(88.5%)	0.02
	Black	51 (5.7%)	46 (4.6%)	11 (5.6%)	729 (6.3%)	837 (6.1%)	
	Other	43 (4.8%)	50 (5.0%)	9 (4.6%)	431 (3.7%)	533 (3.9%)	
	Unknown	9 (1.0%)	5 (0.5%)	4 (2.0%)	182 (1.6%)	200 (1.5%)	
**Hispanic**	Unknown	23 (2.6%)	29 (2.9%)	7 (3.6%)	809 (7.0%)	868 (6.4%)	<0.01
	No	810 (89.9%)	907 (90.4%)	174 (88.8%)	9840 (85.4%)	11731(86.1%)	
	Yes	68 (7.5%)	67 (6.7%)	15 (7.7%)	872 (7.6%)	1022 (7.5%)	
**Tumor location**	NOS	118 (13.1%)	116 (11.6%)	35 (17.9%)	1989 (17.3%)	2258 (16.6%)	< .01
	Cerebrum	764 (84.8%)	884 (88.1%)	159 (81.1%)	9079 (78.8%)	10886(79.9%)	
	Midline	19 (2.1%)	3 (0.3%)	2 (1.0%)	453 (3.9%)	477 (3.5%)	
**Charlson-**	0	774 (85.9%)	850 (84.7%)	168 (85.7%)	9867 (85.6%)	11659(85.6%)	0.76
**Deyo**	1	93 (10.3%)	101 (10.1%)	20 (10.2%)	1190 (10.3%)	1404 (10.3%)	
**Score**	2	23 (2.6%)	38 (3.8%)	7 (3.6%)	358 (3.1%)	426 (3.1%)	
	≥3	11 (1.2%)	14 (1.4%)	1 (0.5%)	106 (0.9%)	132 (1.0%)	
**Facility**	Unknown	518 (57.5%)	443 (44.2%)	105 (53.6%)	5246 (45.5%)	6312 (46.3%)	< .01
**Type**	Community	135 (15.0%)	225 (22.4%)	38 (19.4%)	2960 (25.7%)	3358 (24.7%)	
	Academic	248 (27.5%)	335 (33.4%)	53 (27.0%)	3315 (28.8%)	3951 (29.0%)	
**Living area**	Unknown	26 (2.9%)	34 (3.4%)	4 (2.0%)	439 (3.8%)	503 (3.7%)	0.96
	Metro	730 (81.0%)	797 (79.5%)	152 (77.6%)	9262 (80.4%)	10941(80.3%)	
	Rural	145 (16.1%)	172 (17.1%)	40 (20.4%)	1820 (15.8%)	2177 (16.0%)	
**Treatment**	Unknown	37 (4.1%)	48 (4.8%)	7 (3.6%)	665 (5.8%)	757 (5.6%)	< .01
	None	40 (4.4%)	40 (4.0%)	7 (3.6%)	1022 (8.9%)	1109 (8.1%)	
	RT+CT+S	138 (15.3%)	102 (10.2%)	31 (15.8%)	1184 (10.3%)	1455 (10.7%)	
	RT+CT	41 (4.6%)	36 (3.6%)	12 (6.1%)	825 (7.2%)	914 (6.7%)	
	RT+S	115 (12.8%)	79 (7.9%)	27 (13.8%)	1348 (11.7%)	1569 (11.5%)	
	CT+S	56 (6.2%)	181 (18.0%)	14 (7.1%)	645 (5.6%)	896 (6.6%)	
	S only	417 (46.3%)	444 (44.3%)	92 (46.9%)	4705 (40.8%)	5658 (41.5%)	
	RT only	39 (4.3%)	21 (2.1%)	2 (1.0%)	772 (6.7%)	834 (6.1%)	
	CT only	18 (2.0%)	52 (5.2%)	4 (2.0%)	355 (3.1%)	429 (3.1%)	
**Insurance status**	Not Insured	57 (6.3%)	68 (6.8%)	14 (7.1%)	808 (7.0%)	947 (7.0%)	< .01
	Private	639 (70.9%)	741 (73.9%)	136 (69.4%)	7629 (66.2%)	9145 (67.1%)	
	Medicaid	110 (12.2%)	98 (9.8%)	25 (12.8%)	1236 (10.7%)	1469 (10.8%)	
	Medicare	69 (7.7%)	72 (7.2%)	18 (9.2%)	1374 (11.9%)	1533 (11.3%)	
	Other Gov’t	20 (2.2%)	18 (1.8%)	1 (0.5%)	215 (1.9%)	254 (1.9%)	
	Unknown	6 (0.7%)	6 (0.6%)	2 (1.0%)	259 (2.2%)	273 (2.0%)	

**NOS:** Not otherwise specified; **RT:** Radiation therapy; **CT:** Chemotherapy; **S:** Surgery

Astrocytic histology included 6,050 (44.4%) patients, oligodendroglial included 4,776 (35.1%), and mixed comprised 2,795 (20.5%) patients (Table 2). Gender distribution and Hispanic origin were not different among histology groups. Black race was more common among astrocytic tumors (7.2%). The most common primary site location was the cerebrum (79.9%), followed by “other” (16.6%), and midline (3.5%). 1p/19q co-deletion status was unknown in 9,617 patients (84.6%), reported as co-deleted in 901 (6.6%), non-co-deleted in 1,003 (7.4%), and incomplete in 196 (1.4%) patients (Table 3). 1p/19q co-deletion testing is only available after 2010 with an increasing tendency. Patients with 1p/19q co-deletion were significantly younger than the non-co-deleted or incomplete groups, with a median age of 37 years (p<0.01). Gender and Hispanic distribution were similar among all groups (Table 1, Table 2) in a comparison of molecular factors (p = 0.98). A higher frequency of patients with 1p/19q co-deletion had a midline primary site location compared to those with 1p/19q non-co-deleted (2.1% versus 0.3%, p<0.01).

**Fig 1:**
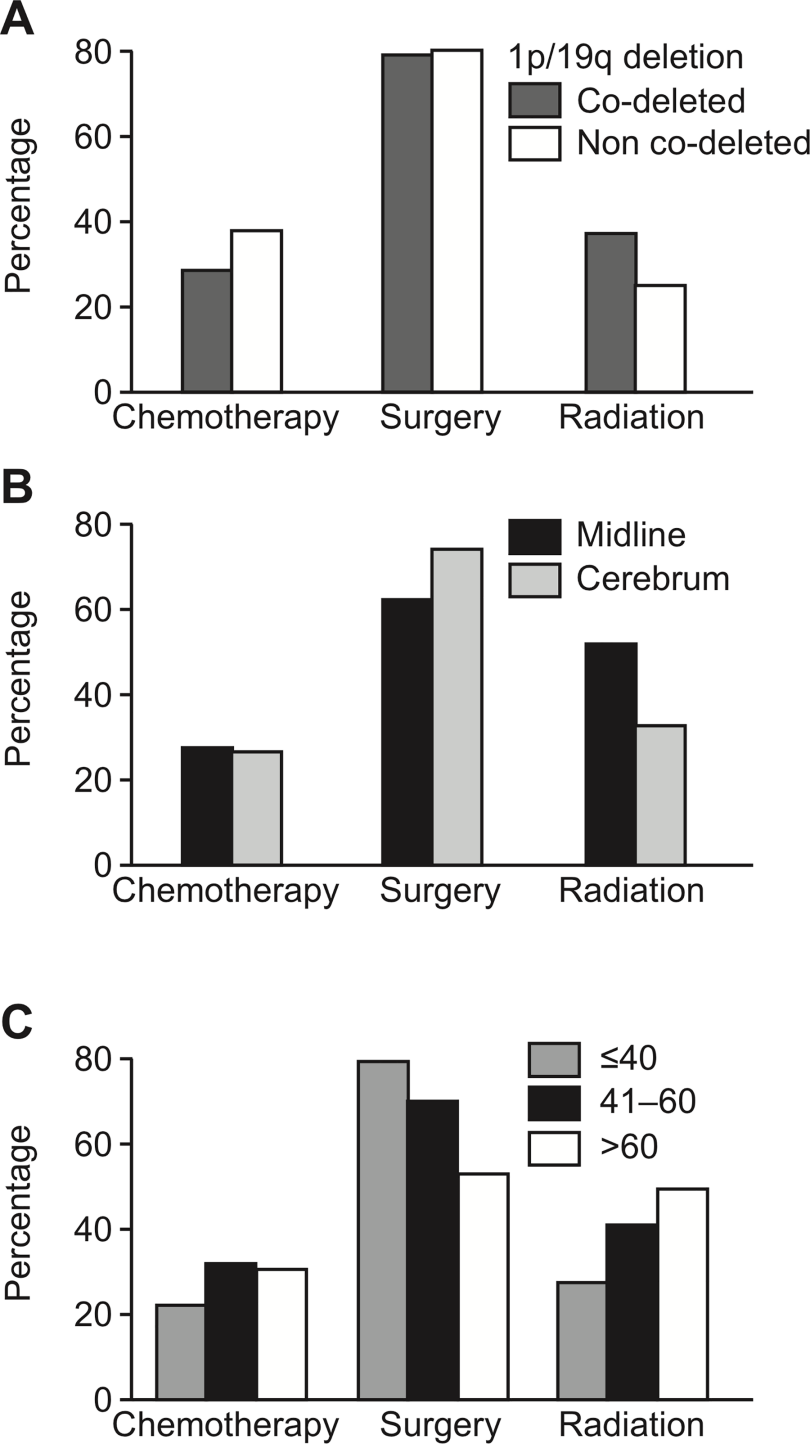
**A.** Chemotherapy, surgery, and radiation treatment receipt by 1p/19q co-deletion status. **B.** Chemotherapy, surgery, and radiation treatment receipt by tumor location. **C.** Chemotherapy, surgery, and radiation treatment receipt by age.

Overall, the most common treatment modality was surgery (72.2%), followed by radiation (36.0%), and chemotherapy (27.3%); 41.5% were treated with surgery only (Fig 1). Chemotherapy and radiation were generally administered as adjuvant treatment; 6.1% of the patients received radiation only and 3.1% received chemotherapy only. The maximal treatment combination (radiation, chemotherapy, and surgery) was used in 10.7% of the patients. Other combinations included radiation and surgery (11.5%), chemotherapy and surgery (6.6%), and radiotherapy and chemotherapy (6.7%). Treatment modality was unknown in 5.6% of the cases, and 8.1% received no treatment.

Treatment varied significantly with age (Table 1, Fig 1). The percent of patients undergoing gross total resection decreased with increasing age. The use of adjuvant treatment was more common in patients older than 40 years of age. Radiation only, radiation plus chemotherapy, and no treatment were more common in patients over 60 years of age. Primary site location was also a main determinant of treatment. Patients with midline lesions were less likely to be treated with surgery alone and more commonly received radiation compared to primary site location in the cerebrum. Interestingly, there was a higher percentage of astrocytic tumors with midline primary site location (6.2%), compared to oligodendroglial tumors (0.8%). Oligodendroglial tumors were also more likely to be located in the cerebrum compared to astrocytic tumors.

Histological classification and molecular signatures had an impact on treatment receipt (Table 2, Fig 1). Astrocytic tumors were more likely to be treated with radiation (43.8% versus 25.3%, p<0.01) while oligodendroglial tumors were more likely to receive surgery (79.9% versus 64.6%, p<0.01). Chemotherapy did not vary between both groups (p = 0.07). Adjuvant treatment was more common in patients with astrocytic histology. Patients with oligodendroglial histology were more likely to be treated with surgery only or chemotherapy plus surgery, and were likely to receive radiation.

Radiation was more frequent in patients with 1p/19q co-deleted than in patients with non-co-deleted status (37.3% versus 24.3%, p<0.01) (Table 3, Fig 1). Chemotherapy was more common in patients with 1p/19q non-co-deleted than in those with 1p/19q co-deleted (37.4% versus 28.1%, p<0.01). Combination therapy with radiation, chemotherapy plus surgery, radiation plus chemotherapy, and radiation plus surgery were more common in patients with 1p/19q co-deleted (15.3%, 4.6%, and 12.8%, respectively). Combined chemotherapy and surgery was more frequent in patients with 1p/19q non-co-deleted (18%).

Socioeconomic factors were assessed by insurance status, income level, and dwelling area. The most common primary payer was private insurance (67.2%), followed by Medicare (11.9%), and Medicaid (10.8%). Government insurance covered 23.9% of patients. The type of primary payer varied with age and histologic groups. The majority of patients older than 60 years of age were covered by a type of government insurance (65.6%); primarily by Medicare (61.8%). Private insurance was the main coverage in patients younger than 60 years while 17.3% were covered by any type of government insurance.

Median annual income was higher than $38,000 per year in 83.7% of patients. Socioeconomic status was somewhat lower in patients with astrocytic tumors with lower income and educational variables. Over 80% of the patients lived in metropolitan areas. The proportion of patients living in rural areas was significantly higher for patients older than 60 years of age. Histology groups were not associated with area of residence. Twenty-nine percent of patients were reported to have received treatment in academic facilities, while 25% were treated in community hospitals. The setting of the facility were patients were treated was unknown in 46.3%.

### Survival

**Fig 2:**
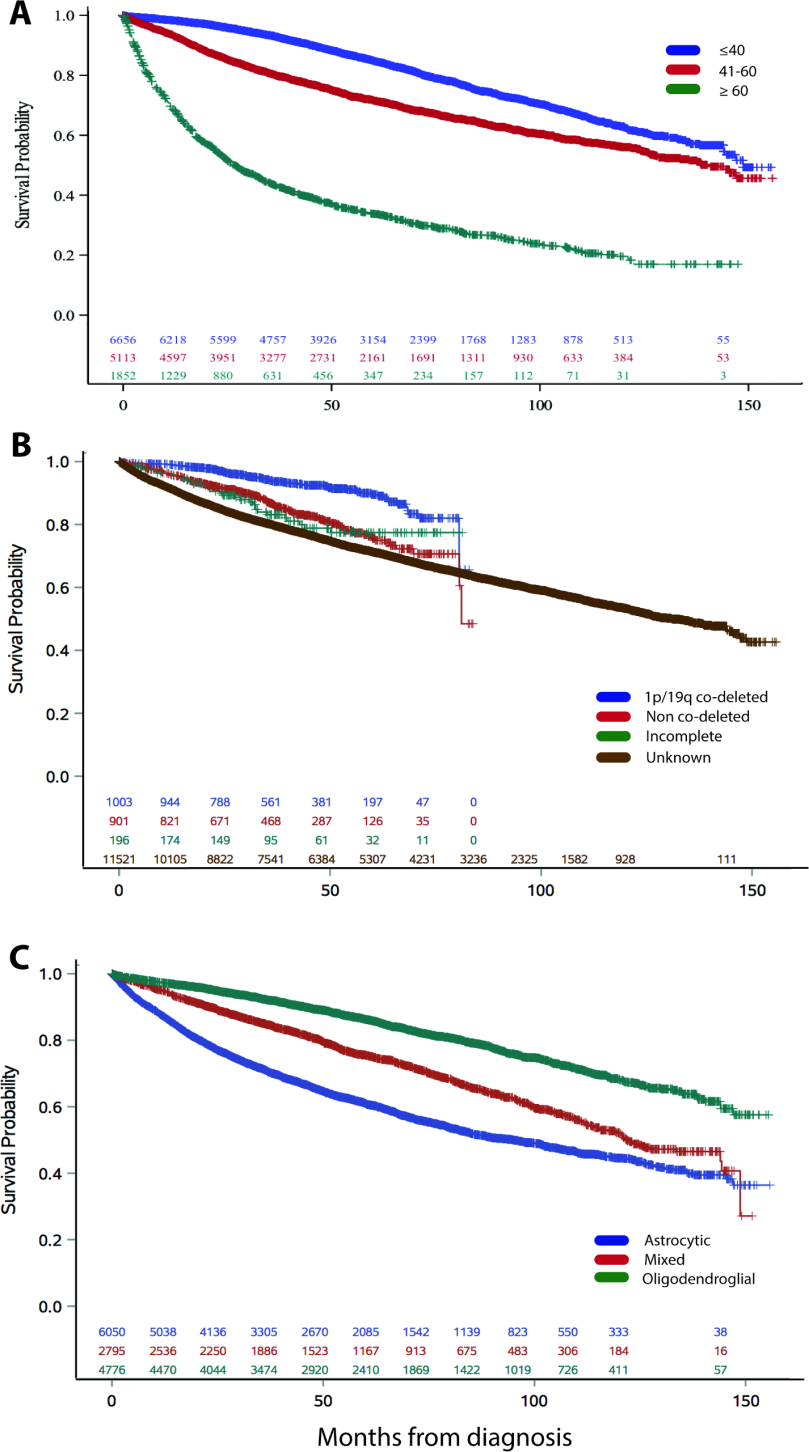
**A.** Survival by age group. **B.** Survival by 1p/19q co-deletion status. **C.** Survival by histology group.

**Fig 3:**
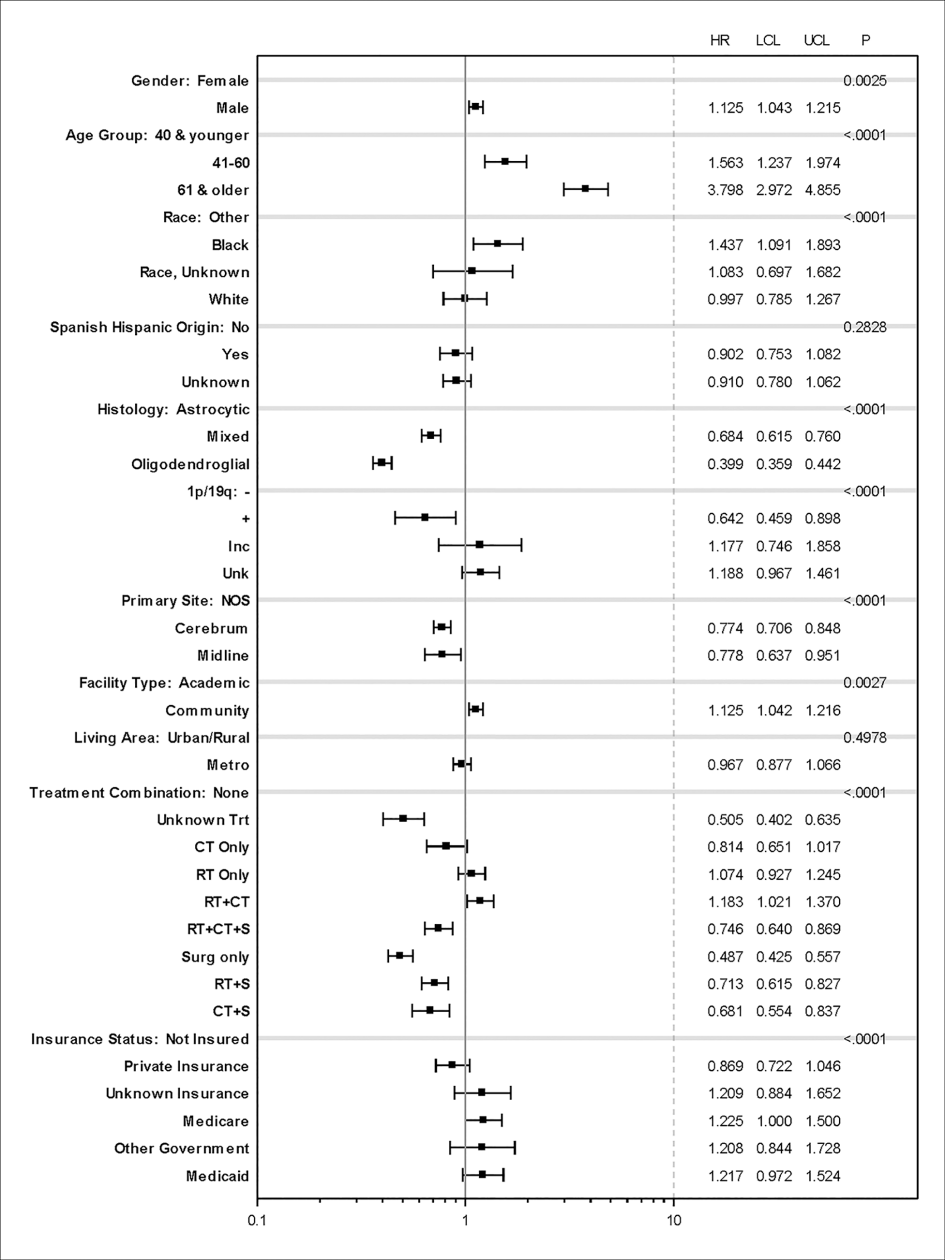
Multivariate analysis for risk factor for mortality.

Median overall survival was 11.3 years. Survival varied by age, comorbidities, tumor location, histology group, and molecular predictors; younger age, fewer comorbidities, oligodendroglial histology, 1p/19q co-deletion, and cerebrum location had the best prognosis (Fig 2, Fig 3). Median survival was greater in patients younger than 60 years of age (12.4 and 11.7 years for <40 and 41–60 years old), compared to 2.2 years in patients older than 60 years. Median survival was not reached in patients with 1p/19q co-deleted low-grade gliomas. Median survival was 7.9 years in astrocytic tumors, 10 years in mixed histology, and was not reached in oligodendroglial tumors.

We performed a multivariate analysis for risk factors for mortality (Fig 3). No treatment, radiation only, and chemotherapy plus radiation were associated with increased risk of mortality. Male gender and black race, as compared to other, were associated with mortality. Medicaid and Medicare, and treatment in community facilities were associated with an increased risk of death as compared to not insured.

## Discussion

With the updated WHO of the nervous system in 2016 molecular profiling is required for proper low-grade glioma classification[1]. Risk assessment is based on three groups: IDH mutant tumors with 1p/19q co-deletion (predominantly oligodendroglial), IDH mutant without 1p/19q co-deletion (predominantly astrocytic), and IDH wild-type tumors[19–21]. The NCDB participant user file from 2004 to 2015 uses the WHO 2007 classification and some molecular markers such as IDH status were not available in our study[16, 22]. Molecular profiling reports were available after 2010 for 15.41% of the patients (Table 3), with an increasing testing tendency.

In NCDB, overall survival was 11 years for all patients evaluated in CoC accredited hospitals in the United States. This is comparable to the 13-year median survival reported by Buckner et al. for high-risk patients receiving chemotherapy and radiation[23], and surpasses the median survival reported in the majority of clinical trials, where it ranges from 7 to 9 years[24, 25]. Age 60 years and younger, 1p19q co-deletion, and oligodendroglial tumors were factors associated with best survival in Kaplan-Meier analyses (Fig 2), and the results held in multivariate proportional hazards models (Fig 3). Tumor location predicts the aggressiveness of treatment due to the risk of functional impairment, that affects long-term survival[26]. Location, such as midline location is also associated with molecular profiling characteristics associated with poor prognosis[8, 27, 28].

Sociodemographic factors that include income, type of insurance, and facility location (academic versus community) are also associated with mortality. Patients treated in community setting had an increased risk of mortality compared to patients treated in academic centers. Similar findings have been described in glioblastoma[29], and suggests a role in access to neuro-oncological care and the role of hospital volume and physician’s expertise in treatment decisions. However, differences in risk factor exposure, disease severity, and population differences may also account for the observed results.

High-risk features for mortality in patients with diagnosis of low-grade gliomas include age older than 40 years, tumor diameter greater than 6 cm, midline crossing, presence of neurological deficit, and astrocytic histology[10]. Shaw *et al*. determined that patients defined as low risk after gross total resection have a 50% risk of tumor progression at 5 years[11]. Based on this, active surveillance remains an option for low risk patients[11]. However, due to the overlapping molecular prognostic factors, heterogeneity of these tumors, and challenges of completing clinical trials in a rarer and long surviving cancer, treatment recommendations remain unestablished.

The longevity associated with these tumors provides concerns for long-term treatment-associated toxicities. Treatment for brain tumors carries the concern for cognitive and functional impairment[30], yet, not receiving treatment allowing for disease progression with associated neurologic deficits and significant mortality.

Our results reflect that patients who receive chemotherapy plus surgery reach comparable survival to those receiving surgery alone. Iwadate *et al*. found similar results in a nonrandomized trial[31], reporting equivalent outcomes in patients who underwent surgery alone versus those who received adjuvant chemotherapy. The benefits of surgery with maximal safe resection are established[32]. Our study with over 70% receiving surgery as first course of treatment demonstrates the benefit of this and is in keeping with the literature, as surgical resection and the extent of the resection has a significant survival benefit[33–35].

The recommendation of chemotherapy as adjuvant treatment is based on the extent of resection, molecular features, and general state of patient. Chemotherapy is an option in patients with a favorable molecular profile, and as a way to delay or forgo radiation[36]. Acknowledging gliomas as a wider brain involved disease than based on imaging, and that the majority of tumors will progress without further treatment[37], the use of chemotherapy is an option for selected high-risk patients to delay disease progression.

Triple combination therapy of surgery plus chemotherapy plus radiation was uncommon (10.7%) but was associated with survival benefits. Retrospective analysis have shown similar results[38]. Clinical trials have demonstrated that combination procarbazine, lomustine and vincristine plus radiation improves outcomes in high risk patients[23, 25]. The type of chemotherapy agent received is not detailed in NCDB and is a limitation to NCDB analysis. Procarbazine, lomustine and vincristine (PCV) combination or temozolomide are the most used regimens[39], and are thought to be comparable. Adjuvant temozolomide have demonstrated survival benefits in anaplastic gliomas and high-risk low grade gliomas[39, 40].

Chemotherapy alone did not demonstrate survival benefits when compared to radiation alone, or radiation plus chemotherapy in a phase III randomized clinical trials[41, 42], but was associated with survival benefits in our analysis. Single center experiences and retrospective analyses in unresectable tumors have shown some benefits[43, 44]. Chemotherapy alone may be of benefit in selected patients in whom resection is not feasible.

The use of radiation alone and radiation plus chemotherapy was associated with increased risk of mortality. Radiation use was significantly higher in patients with 1p/19q co-deleted, older age, and astrocytic histology. These patients may have had less proper tumor resective surgery due to their age and disease burden, and/or a more aggressive clinical presentation. As to why 1p/19q co-deletion are more associated with radiation, it is possibly due to late disease presentation, or being more aggressive on a treatment responsive cancer. Although analyzing different clinical trials are problematic, the upfront radiation alone trials without chemotherapy have median survival rates that are less than a decade[39, 45, 46]. Early radiation has not shown to improve overall survival in randomized trials compared to patients who receive radiation at the time of progression[45]. The use of radiation is associated with significant adverse events, mainly neurocognitive deterioration, vasculopathies, and secondary malignancies[30, 47], and remains a topic of discussion as first line treatment.

NCDB provides long-term follow up and quality data on first course of treatment, but does not include treatment associated complications, or cause of death. Molecular profiling and tumor characteristics beyond our study are not widely available in the data, as well as individual patient assessments. However, our investigation provides an unique opportunity to evaluate patterns of care across the largest cancer registry[15].

In summary, outcomes for low-grade gliomas in most of United States is at or beyond reported in clinical trials and the use of adjuvant therapy may be associated with survival benefits in selected high-risk patients. The role of radiation remains under investigation as first course of treatment in all low-grade gliomas. Outcomes will continue to improve with further understanding of tumor biology and behavior, improving patient selection.

## Supporting information

S1 Fig Multivariate analysis for determinants of treatment receipt comparing patients who received surgery alone with patients that received surgery and adjuvant treatment (either surgery plus chemotherapy, surgery plus radiation, or surgery plus chemotherapy and radiation). (TIF)
